# Management of locally-advanced dermatofibrosarcoma protuberans: Four cases report

**DOI:** 10.1097/MD.0000000000047778

**Published:** 2026-03-06

**Authors:** Haoran Zhang, Lu Zhang, Huaisheng Wang

**Affiliations:** a Department of Burn and Plastic Surgery, West China Hospital, Sichuan University, Chengdu, China; b Department of Dermatology, West China Hospital, Sichuan University, Chengdu, China.

**Keywords:** dermatofibrosarcoma protuberans, imatinib neo-adjuvant therapy, Mohs surgery, wide local excision

## Abstract

**Rationale:**

Dermatofibrosarcoma protuberans (DFSP) is a malignant skin tumor originated from of fibroblast, which remains a challenge in terms of diagnosis and treatment, especially in those with locally-advanced disease.

**Patient concerns:**

Case 1 is a 38-year-old male complained with a mass at his left temporal and left mandibular region, and invaded his left orbital region. Case 2 is a 54-year-old male with a mass located in this chest region. The tumor invaded 2th and 3rd ribs with suspicious intra-thoracic invasion. Case 3 is a 63-year-old male with a recurrent tumor in abdominal wall which invaded peritoneum of abdominal wall. Case 4 is a 11-year-old boy with a mass located in his left forearm, which invaded the abductor pollicis longus, extensor pollicis brevis, and extensor carpi radialis longus.

**Diagnoses:**

These 4 patients were diagnosed with DFSP with local invasion. Case 3 was pathologically diagnosed with reginal fibrosarcomatous transformation (FS-DFSP).

**Interventions:**

Case 1 received imatinib 400 mg/day as neo-adjuvant therapy. After 6 months, the mass significantly shrunk and Mohs surgical resection and reconstruction with free anterolateral thigh flap were performed. Case 2 received Mohs surgical resection and reconstruction with contralateral pedicled latissimus dorsi flap. Case 3 and Case 4 also received Mohs surgical resection of the tumor and construction with a free anterolateral thigh flap.

**Outcomes:**

All of the 4 patients achieved clean margins by frozen section during surgery. At the last follow-up, none of them experienced disease recurrence.

**Lessons:**

Mohs micrographic surgery is an effective surgical technique for locally-advanced DFSP patients. Imatinib neo-adjuvant therapy is an option when surgical resection with negative margin may cause unacceptable functional or cosmetic outcomes.

## 1. Introduction

Dermatofibrosarcoma protuberans (DFSP) is an uncommon, low-grade sarcoma of fibroblast origin with an incidence rate of 6.25 cases per million person-years.^[1]^ The initial clinical manifestation of DFSP is usually a painless, slowly growing, violaceous nodule or plaque.^[2]^ More than 85% of DFSPs are low-grade tumors with slow growth rate over a period of months to years, with low rates of regional or distant metastasis.^[3]^

Histologically, DFSP originates within the dermal layer of the skin and gradually involves the subcutaneous tissue and beyond. In long-standing cases, it invades the underlying fascia, muscle, and even bones.^[4]^ Three-dimensional reconstruction of DFSP has revealed tumors with highly irregular shapes and frequent finger-like extensions.^[5]^ Thus, surgical removal with wide local excision (WLE) or Mohs micrographic surgery (MMS) technique of DFSP is now recommended for patients with locally-confined DFSP.^[6,7]^ However, due to its mimicking clinical presentations with other benign skin tumors, delay in diagnosis is not uncommon DFSP patients, and some of them presented with large tumor size and locally invasion at the time of diagnosis.^[8]^ For these patients, completely surgical resection and reconstruction has always been a challenge for surgeons.

Here we presented 4 cases of locally-invasive DFSP with challenging resection and reconstruction in head, chest, abdominal regions, and extremities. The aim of this study is to discuss the resection and reconstructive options for locally-advanced DFSP patients.

## 2. Case report

### 2.1. Case 1: DFSP in the head region

**Figure 1.**
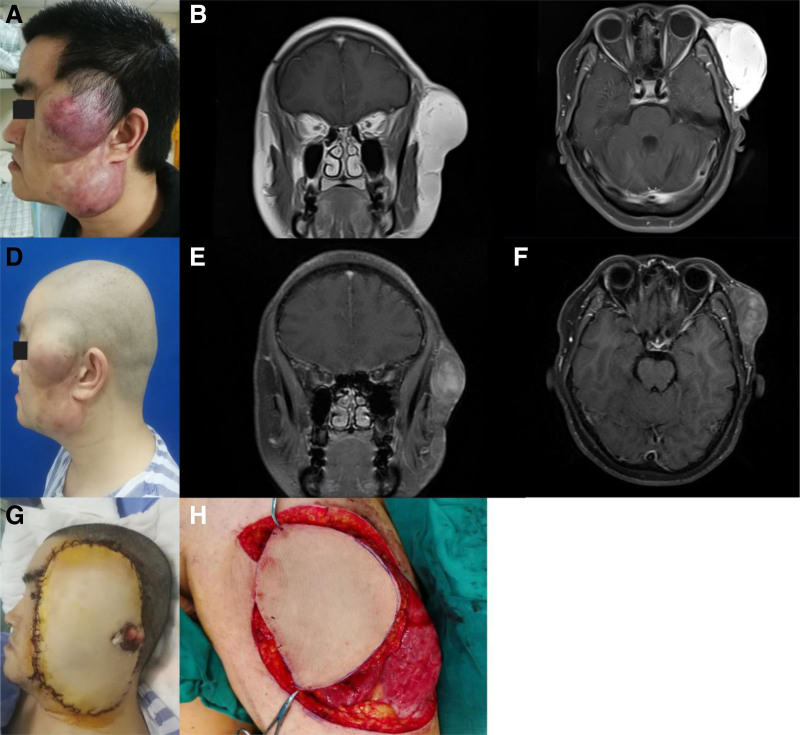
Clinical presentation and MRI findings of case 1. (A) DFSP lesion located in the face at baseline; (B–C) Magnetic resonance imaging prior to the beginning treatment with imatinib mesylate; (D) DFSP lesion after treatment with imatinib mesylate; (E–F) Magnetic resonance imaging after treatment with imatinib mesylate; (G) Surgical region after resection and reconstruction using free latissimus dorsi flap; (H) Harvested free anterolateral thigh flap.

A 38-year-old male came to hospital for his left temporal mass in Nov. 2019. He underwent surgical resection of left temporal mass 6 years ago and histologically diagnosed as DFSP. Fluorescence in situ hybridization analysis suggested COL1A1/PDGFB gene fusion and PDGFB gene translocation. No adjuvant therapy was administered since last surgery. He experienced disease recurrence as a solid, subcutaneous lobular mass at his left temporal and left mandibular region one year ago, and the tumors gradually grew into 20 × 10cm and invaded his left orbital region (Fig. 1A–C). The case was discussed in our multi-disciplinary treatment group, and imatinib 400 mg/day was administrated as neo-adjuvant therapy. The patient only experienced mild nausea during treatment. After 6 months of targeted therapy, the mass shrunk into 12.8 × 5.5 × 2.8cm measuring by magnetic resonance imaging (MRI) (Fig. 1D–F). No enlarged lymph nodes in the neck region were reported.

Surgical resection with Mohs micrographic surgery technique was performed. Patients’ left auricle and parotid gland were also resected during surgery. Gross finding of the excised mess was 2 predominant tumors with the size of 5 × 5 × 3.5cm in the temporal region, and 4.5 × 4.5 × 3.0cm in the mandibular region. Intraoperative frozen section suggested no involvement of surgical margin. A free anterolateral thigh flap was harvested from the thigh and microvascular anastamosis was performed between the flap vasculature (cutaneous perforators of the descending branch of the lateral femoral circumflex vessels) and the left facial vessels (Fig. 1G–H). Pathological findings of the paraffin specimen confirmed DFSP. Immunohistochemical studies of EMA, S-100, desmin, SMA, Calponin, and CD10 were all reported as negative. CD34 was confirmed as positive, and the Ki-67 proliferation index was 5%. No radiotherapy was administrated after last operation. In the latest follow-up 5 years after surgery (Oct. 2024), the flap is well healed and the patient is disease free.

### 2.2. Case 2: DFSP in the chest region

**Figure 2.**
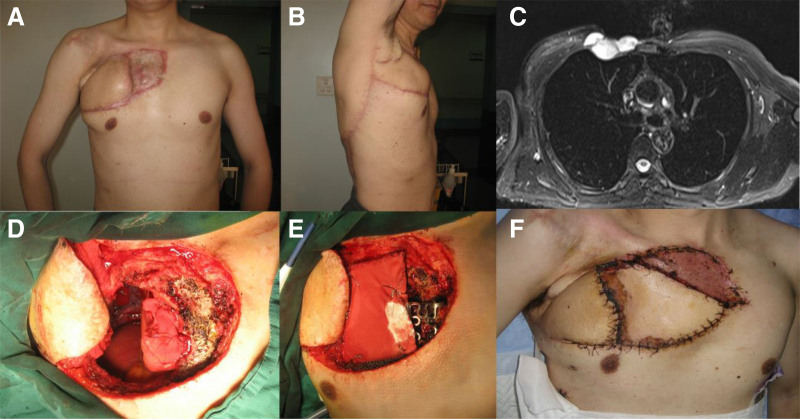
Clinical presentation and MRI findings of case 2. (A–B) DFSP lesion located in the chest region; (C) Magnetic resonance imaging before surgery; (D) 2nd and 3rd ribs and adjacent pleura were resected during surgery; (E) Chest wall defect was covered by titanium and polyester meshes; (F) Immediate post-operative picture with the flap sutured to the chest wall defect.

A 54-year-old male presented in Dec. 2016 with a complaint of recurrent mass in the right chest region for 6 months. In his 20s, he received surgical resection of a tumor on his right chest region, which later pathologically confirmed to be DFSP. Then he experienced 3 times of disease recurrence and surgery, without any adjuvant therapy after surgery. Six months ago, he once again noticed a mass in the right chest region. Physical examination suggested a painless mass in his right chest region. Clinically the mass was 5 × 5 cm involving the skin and subcutaneous tissue with prominent hatch marks around ipsilateral latissimus dorsi flap (Fig. 2A–B). MRI exam revealed a hypodense, irregular mass measuring 4.8 × 3.6 × 2.3cm closely in front of the 2th and 3rd ribs with suspicious intra-thoracic invasion (Fig. 2C). No enlarged lymph nodes in the neck and axillary regions were reported. No lung nodules and enlarged mediastinal nodes were identified from the computed tomography scan of the chest.

Surgical resection was performed with a wide margin of 1cm. The gross finding of the excised mess was a 5.3 × 5.2 × 2.5cm sized tumor located in the right 2nd and 3rd anterior intercostal space, and the 2nd and 3rd anterior ribs and part of the right sternum were invaded. The tumor did not invade pleura and pericardium (Fig. 2D). Serial sections of the gross specimen revealed a tan white, lobulated, and rubbery mass, with scattered areas of necrotic tissue. Frozen section during surgery confirmed clean margins of the operative area. Two pieces of titanium mesh and one piece of polyester mesh (Dacron) were used to cover the defect of right chest wall (Fig. 2E). Contralateral pedicled latissimus dorsi flap was prepared and rotated to the right chest wall through the subcutaneous tunnel. Secondary defect was covered with split-thickness skin graft from the left thigh. Secondary skin grafting from the left thigh to right chest wall was performed due to unsatisfying blood supply at the distal end of the flap (Fig. 2F).

At the 3-month postoperative follow-up visit since the initial surgery, the patient has recovered well without any postoperative complications. No local or systemic tumor recurrence was detected from physical examinations and MRI scan. The patient remains disease free at latest follow-up at Oct. 2024.

### 2.3. Case 3: DFSP in the abdominal region

**Figure 3.**
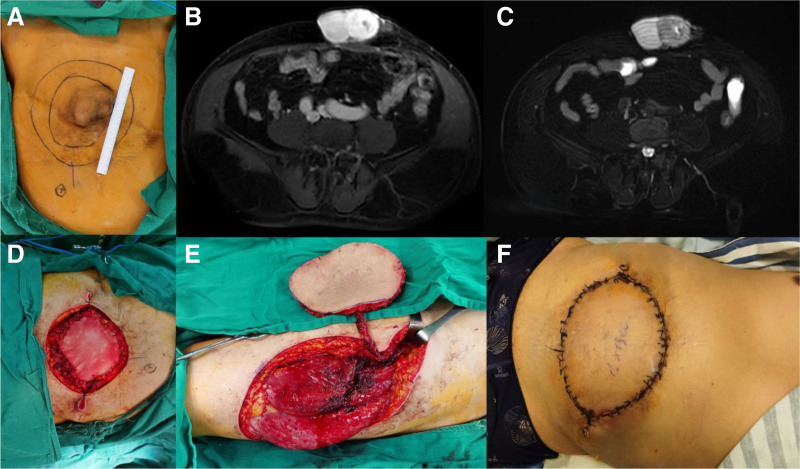
Clinical presentation and MRI findings of case 3. (A) DFSP lesion located in the abdominal region; (B–C) Magnetic resonance imaging before surgery; (D) tumor and adjacent abdominal muscles and peritoneum were resected, and polypropylene mesh was used to cover the defect; (E) free anterolateral thigh flap was harvested; (F) Immediate post-operative picture with the flap sutured to the abdominal wall defect.

A 63-year-old male presented with a recurrent abdominal wall tumor came for medical consultation in Feb. 2021. The patient received an abdominal wall tumor resection 23 years earlier and pathologically diagnosed as DFSP. Clinically the mass was 10 × 8 cm involving the skin and subcutaneous tissue with a 12 × 8cm scar region from previous surgery. MRI of the abdominal region revealed a 9.5 × 6.8 × 4.2 cm dense and hyperintensity mass at the anterior lower abdominal wall, and closely adjacent to the blood vessels, muscles, and peritoneum of abdominal wall (Fig. 3A–C). No obvious intra-abdominal organs were involved, and no lymph nodes in the abdominal region were reported from MRI.

The abdominal mass was surgically removed with a circumferential margin of 3 cm, and the involved peritoneum was also resected. The gross finding of the excised mass was a 7.5 × 6.8 × 4.2 cm subcutaneous mass. Serial sections of the gross specimen revealed a tan white, lobulated, solid, medium hardness mass. Frozen section during surgery confirmed clean margins and base of the operative area. Pathological findings confirmed the tissue as DFSP Grade 2 (Fédération Nationale des Centres de Lutte Contre le Cancer grading system) with regional fibrosarcomatous transformation (FS-DFSP). One piece of polypropylene mesh was used to cover the abdominal wall defect. Multiple pedicled fasciocutaneous flaps were used to shrink the defect area (Fig. 3D). A free anterolateral thigh flap was harvested from the thigh and microvascular anastamosis was performed between the flap vasculature (cutaneous perforators of the descending branch of the lateral femoral circumflex vessels) and the left inferior epigastric vessels (Fig. 3E and F). Secondary defect was covered with split-thickness skin graft from back of the left thigh.

At the 3-month postoperative follow-up visit since the initial excision of the mass, the patient has recovered well without any postoperative complications. At the latest follow-up visit in Oct. 2024, no local or systemic tumor recurrence was detected from physical examinations and MRI scan.

### 2.4. Case 4: DFSP in the extremities

**Figure 4.**
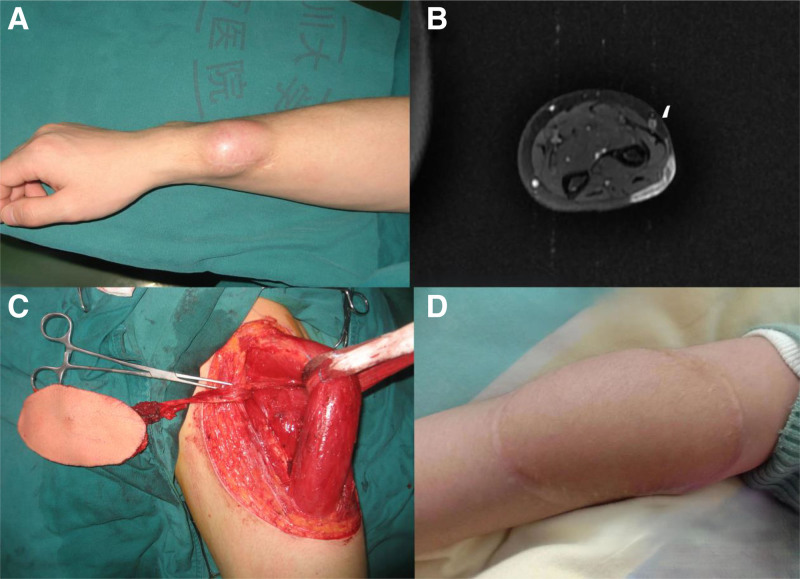
Clinical presentation and MRI findings of case 4. (A) DFSP lesion located in the forearm; (B) Magnetic resonance imaging before surgery; (C) free anterolateral thigh flap; (D) post-operative picture.

A 11-year-old boy came to visit with a mass at his left forearm in Oct. 2021. Initially, the mass grew from a cat-scratch wound, and was gradually progressive in size, measuring around 3 cm in diameter. The patient denied any pain at the left forearm. Physical examination indicated a skin-colored, fixed 3 × 2 cm mass with no skin necrosis and ulceration at the left forearm. The mass was soft tissue-like in consistency. No other significant physical finding was found. A MRI of his left arm was performed, which showed a 3.1 × 2.4 × 0.5cm heterogeneous density mass at the distal part of the left forearm with suspicious invasive of abductor pollicis longus, extensor pollicis brevis, and extensor carpi radialis longus. Left ulna and radius were not involved (Fig. 4A and B).

Surgical resection with circumferential margin of 1cm was applied, and the involved muscle was also resected. Gross finding of the excised mass was 2.8 × 2.4 × 0.4 cm in size. Frozen section during surgery confirmed clean margins and base of the operative area. Immunohistochemical studies COL1A1/PDGFB gene fusion and PDGFB gene translocation. A free anterolateral thigh flap was harvested from the thigh and microvascular anastamosis was performed between the flap vasculature (cutaneous perforators of the descending branch of the lateral femoral circumflex vessels) and the left radial vessels. Secondary defect was covered with split-thickness skin graft from back of the left thigh (Fig. 4C).

At the 3-month postoperative follow-up visit since the initial wide excision of the mass, the patient has recovered well without any disease recurrence. At the latest follow-up visit in Nov. 2024, no local or systemic tumor recurrence was detected from physical examinations and MRI scan (Fig. 4D).

## 3. Discussion

DFSP is a rare, slow-growing, locally aggressive tumor.^[9]^ No definitive risk factors for the development of DFSP have been identified. However, DFSP is most commonly seen in the 30s to 50s years old populations. Besides, DFSP most commonly arises in the trunk (42%), upper extremities (23%), lower extremities (18%), and head and neck (13%)^[10]^ DFSP often presented with a fairly innocuous gross appearance, which leads to high incidence of misdiagnose. In this article, we presented 4 DFSP cases located in head, chest, abdomen, and extremities, separately, and presented resection and reconstruction options for individual patient. Morphological studies of DFSP have revealed highly irregular borders with fingerlike extensions into surrounding and deep tissues.^[11]^ Incidence of local recurrence after an inadequate primary resection ranged from 40% to 60%.^[12,13]^ Thus, Complete surgical excision of DFSP is crucial for DFSP patients. WLE and MMS are preferred surgical techniques for DFSP. In 2012, Foroozan et al conducted a meta-analysis including 23 trials with over 600 patients to compare WLE and MMS in DFSP.^[14]^ They reported lower recurrence in patients treated with MMS (1.11%, 95% CI: 0.02–6.03%) compared with WLE (6.32%, 95% CI: 3.19–11.02). Thus, in the National Comprehensive Cancer Network guideline version 1.2024 recommends Mohs or similar techniques over WLE. However, WLE is now more often considering over MMS in many institutions. Some of the reasons could be that frozen sections may not always be available. MMS is a more time-consuming technique than WLE because of repeat resection and frozen section exam. Another major reason is that accurate MMS frozen examination requires a pathologist with specialized training. These factors limit MMS for widely use. Standard WLE with a width of 1 cm around the primary tumor would have left residual microscopic tumor in more than 70% of patients; width of 2 cm, 40%; 3 cm, 15.5%; and 5 cm, 5%. In this study, we used surgical resection technique with extended margins and intraoperative frozen section.^[15]^ In patients which wide resection will cause unacceptable functional or cosmetic outcomes (case 1 and 4), we performed surgery with extended margin of 1 to 2 cm and alongside the tumor in orbital region. As for tumors located in the chest and abdominal regions, resection was performed with circumferential margin of 3cm. Intraoperative frozen section confirmed clean margins for all patients. Both MMS and WLE often results in large defects, especially in locally-advanced patients. Reconstruction of DFSP surgical defects should be delayed until negative margins are confirmed. In some institutions without sufficient intraoperative frozen section technique, delayed wound management has become an effective strategy after clean margin was confirmed by paraffin pathological examination.^[16–18]^ In our cases, frozen section examination for checking peripheral and deep margins was applied during surgery. After tumor-free margins were confirmed during surgery, we immediately performed reconstruction by using free latissimus dorsi flap, free anterolateral thigh flap, and pedicled latissimus dorsi flap to cover the defect area.

DFSP is characterized by either a genomic rearrangement involving chromosomes 17 and 22, in a supernumerary ring chromosome containing chromosomes 17 and 22 sequences, or in a reciprocal balanced translocation t (17; 22) (q22; q13).^[19,20]^ It leads to the formation of the collagen type I-alpha 1/platelet-derived growth factor beta (COL1A1–PDGFB) fusion gene.^[21]^ This fusion gene could be activated through an autocrine loop of the PDGF-β, which is a potent mitogen for mesenchymal cells that activates the Ras MAPK and PI3K-AKT-mTOR pathways, leading to cell growth and differentiation^[4,22]^ The discovery of dysregulated expression of PDGF-β in DFSP led to the use of tyrosine kinase inhibitors, such as imatinib mesylate, to treat patients with unresectable and metastatic DFSP^[23,24]^ Several studies have shown the benefit of neoadjuvant imatinib for reducing tumor size and achieving complete surgical resection^[25,26]^ Imatinib is a targeted drug that acts on PDGF, mainly used in hematologic tumors. It has been applied to the treatment of unresectable, recurrent and/or metastatic DFSP. McArthur et al believe that the following situations may require imatinib treatment: local advanced unresectable DFSP patients; local advanced DFSP patients, reducing tumor volume can facilitate surgical resection; and metastatic disease not suitable for surgical resection patients. Two phase II clinical trials of imatinib mesylate in advanced DFSP showed about 50% advanced DFSP patients achieved objective response after receiving imatinib treatment^[26]^ The article by Navarrete-Dechent et al shows that for locally advanced DFSP patients, neoadjuvant treatment with imatinib enabled 60% of patients to have the opportunity for surgical treatment^[27]^ In metastatic DFSP patients, 70% of them had tumor shrinkage from imatinib treatment, with median progression-free survival of 11 months^[28]^ In case 1, the patient experienced a significant reduction in tumor volume after receiving imatinib treatment for 6 months. Surgical resection with extended margin of 1cm and alongside the tumor in orbital region achieved a clean margin, which led to satisfying functional and cosmetic results after surgery.

Approximately 80% to 90% of DFSPs are low-grade lesions, whereas the remaining 10% to 20% of DFSPs contain a high-grade fibrosarcomatous (FS-DFSP) component. Evidence suggested that compared with DFSP, FS-DFSP is connected with increased risk of local recurrence, lower time to recurrence, and increased risk of metastasis.^[29–32]^ A systematic review of 1422 patients with DFSP and 225 with DFSP-FS reported risks of local recurrence (29.8% vs 13.7%), metastasis (14.4% vs 1.1%), and death (14.7% vs 0.8%) from the disease to be significantly higher in FS-DFSP versus DFSP.^[33]^ Currently, there is no recommended treatment options for FS-DFSP besides surgery. In case 3, regional fibrosarcomatous transformation was detected by paraffin pathological examination, and this patient did not receive any treatment after surgery and remains disease free for 43 months. Evidence showed that imatinib still shows beneficial for FS-DFSP patients. By analyzing 21 pre- and post-treatment FS-DFSP samples, Tazzari et al discovered that the tumor site during IM therapy. Gene expression profile and immunohistochemistry analyses documented the occurrence of IM-induced senescence phenotype in the tumor cells and showed the accumulation of activated CD3^+^T cells and CD163^+^CD14^+^ myeloid cells expressing the CD209 marker in post-therapy lesions.^[34]^ Rutkowski et al analyzed the data of 31 patients with locally advanced/initially inoperable and/or metastatic DFSP.^[35]^ In patients with FS-DFSP, the 5-year PFS rate was 33%, and 50% patients achieved partial responses.

DFSP is a radio-sensitive tumor. In a single-institution retrospective study of 53 patients treated with surgery and preoperative or postoperative RT, local control was 93% and actuarial overall survival was 98% at 10 years.^[36]^ A systematic review including 22 studies suggested that the local control after postoperative radiotherapy was excellent (75–100%), with a median follow-up time of 69 months.^[37]^ Thus, the National Comprehensive Cancer Network panel is now recommended as adjuvant therapy can be considered for the treatment of positive margin if not given previously and further resection is not feasible.

## 4. Conclusions

Mohs micrographic surgery is an effective surgical technique for locally-advanced DFSP patients. Imatinib neo-adjuvant therapy is an option when surgical resection with negative margin may cause unacceptable functional or cosmetic outcomes.

## Acknowledgments

We would like to extend our deepest gratitude to all the patients who generously participated in this study. Their willingness to share their experiences, and provide valuable clinical data has been the cornerstone of this study.

## Author contributions

**Conceptualization:** Haoran Zhang, Huaisheng Wang.

**Data curation:** Haoran Zhang, Huaisheng Wang.

**Formal analysis:** Haoran Zhang, Huaisheng Wang.

**Investigation:** Haoran Zhang, Huaisheng Wang.

**Methodology:** Haoran Zhang, Lu Zhang, Huaisheng Wang.

**Resources:** Haoran Zhang.

**Software:** Haoran Zhang, Lu Zhang.

**Validation:** Haoran Zhang, Lu Zhang.

**Visualization:** Lu Zhang.

**Supervision:** Huaisheng Wang.

**Writing – original draft:** Haoran Zhang, Lu Zhang.

**Writing – review & editing:** Haoran Zhang, Lu Zhang, Huaisheng Wang.
